# A Quality Improvement Initiative to Alleviate Pain in Flexible Cystoscopy and Minimize False Passages in Catheterization

**DOI:** 10.7759/cureus.104132

**Published:** 2026-02-23

**Authors:** Sheldon P Jolie

**Affiliations:** 1 Urology, Glasgow Royal Infirmary, Glasgow, GBR

**Keywords:** bladder, catheterization, discomfort, flexible cystoscopy, lubricant, pain, prostate, urethra

## Abstract

Introduction: Flexible cystoscopy is a common diagnostic procedure used to evaluate the urethra, bladder, and prostate by inserting a camera-equipped flexible tube through the urethra. Similarly, urinary catheterization involves the passage of a tube into the bladder for drainage or treatment. Both procedures are known to cause discomfort due to urethral sensitivity and share similar pre-procedural preparation steps. This quality improvement project (QIP) aimed to reduce patient discomfort during flexible cystoscopy by improving urethral preparation techniques, with potential applicability to urinary catheterization to minimize traumatic insertions and false passages.

Methods: A mixed-methods approach was used. Observations were made of healthcare professionals performing pre-procedural urethral preparation to identify variations in lubrication techniques. Patient feedback was collected using the Numeric Pain Rating Scale (NPS) and the Visual Analog Scale (VAS) following flexible cystoscopy. After analyzing the findings, the staff was educated on appropriate lubrication techniques, emphasizing adequate gel application prior to instrumentation.

Results: Initial observations revealed that inadequate lubrication was commonly practiced, contributing to increased discomfort during flexible cystoscopy. Questionnaire data confirmed higher reported pain scores among patients who received suboptimal lubrication. Following implementation of proper lubrication techniques, patients reported lower pain and discomfort levels, indicating improved procedural tolerance and patient satisfaction.

Discussion and conclusion: Optimizing the urethral lubrication technique significantly reduces pain during flexible cystoscopy and may prevent traumatic catheterizations. Based on these findings, an instructional video will be developed for the National Health Service (NHS) Greater Glasgow and Clyde (GGC) to demonstrate correct versus incorrect lubrication methods. This initiative is expected to standardize best practices, enhance patient comfort, and reduce complications during urological procedures.

## Introduction

Flexible cystoscopy has been around since 1973, when it was invented by Tsuchida and Sugawara, but it was not fully commercialized until the 1980s. Meanwhile, urinary catheters have been used since at least 3000 BC [1,2]. The rigid cystoscope existed before the flexible cystoscope. The rigid cystoscope allows for direct visualization of the male and female urethra, the prostate in men, and the bladder in both men and women. However, the rigid cystoscope, which existed for a few decades before the flexible cystoscope, had a significant limitation: the discomfort and pain that patients felt during the passage of the scope via the urethra into the bladder [3].

The flexible cystoscope was invented to address this limitation. It was initially designed as a less painful alternative to the rigid cystoscope, and it also allows for a view of the bladder neck, which was previously impossible to achieve. However, over time, the essence of the flexible cystoscope as a less painful cystoscopy method has been lost in today’s practice, and patients (men, in particular) experience significant discomfort and pain during the procedure.

Similar to flexible cystoscopy, urinary catheterization (using a Foley catheter) involves the insertion of a flexible tube of comparable size (16 French (Fr)) to the flexible cystoscope (15.3 Fr) into the bladder to drain fluids or deliver treatment [3]. Both procedures involve the passage of a foreign body into the highly sensitive urethra, and the pre-procedural preparation is almost identical [4]. 

In modern healthcare, the placement of a flexible cystoscope or Foley catheter is understood to be uncomfortable but should not cause significant discomfort or pain. However, after observing men who were visibly uncomfortable or even distraught while undergoing these two procedures, I decided to undertake this quality improvement project (QIP). This study examines the causes of pain and discomfort experienced by men during flexible cystoscopies. It also explores potential modifications to minimize this discomfort and achieve better outcomes.

## Materials and methods

Patients

The patients eligible for this study were men undergoing flexible cystoscopy. The reasons for the flexible cystoscopies included both diagnosis and monitoring. In some cases, transurethral laser ablation (TULA) was performed as an additional procedure. However, the purpose of the procedure did not contribute to study eligibility. The primary focus of the study was pre-procedural preparation. All men above the age of 18 were eligible for this study. Patients with a known psychiatric history were included; however, this contributed to the outliers observed in both groups, as noted in the results. 

Lubricant 

Instillagel (Farco-Pharma GmbH, Cologne, Germany) is a sterile, water-soluble gel formulation containing lidocaine hydrochloride (2%) as a local anaesthetic to provide mucosal analgesia and chlorhexidine gluconate (0.25%) as a broad-spectrum antiseptic to reduce microbial contamination during instrumentation. The preparation also includes methyl hydroxybenzoate (E218) and propyl hydroxybenzoate (E216), which serve as antimicrobial preservatives. The active constituents are suspended in a gel base comprising hydroxyethylcellulose (gelling agent), propylene glycol (solvent and humectant), and purified water, providing optimal viscosity and mucosal adherence for ease of application. This formulation ensures local anaesthesia, antisepsis, and lubrication during urological and other transurethral procedures. Instillagel is supplied in pre-filled sterile syringes containing either 6 mL or 11 mL of gel. The 11 mL presentation is typically used in male patients, whereas the 6 mL syringes are used in female patients during procedures such as Foley catheter insertion and flexible cystoscopy, among other urological interventions. This prepackaged delivery system facilitates aseptic administration and ensures accurate dosing for patient safety and procedural efficiency [5].

Procedure

The procedure for flexible cystoscopy follows a similar preparation technique to that of urethral catheterisation. Two techniques were performed during this study. One, the standard technique performed by urology consultants, where the penis was held taut, and the urethral meatus was cleaned and sterilised. Then, Instillagel was applied to the urethra, and the flexible cystoscope was introduced into the urethra. In the case of a flexible cystoscopy, a comprehensive diagnostic check or other procedure will be performed [6]. Two, an unstandardised technique (typically observed in the clinic) performed by other healthcare professionals (including nurses and trainees), where the penis was not held taut; however, the urethral meatus was cleaned and sterilised in the same manner. Instillagel was then applied to the urethra, and the flexible cystoscope was introduced into the urethra. 

Data collection

A mixed-methods approach was applied using the following techniques. The pre-procedural preparation techniques employed by different healthcare professionals at Glasgow Royal Infirmary (GRI), Glasgow, Scotland, either on the ward (Foley catheterisations) or in the flexible cystoscopy hubs (an outpatient setting where patients can get a diagnostic or monitoring cystoscopy relatively quickly outside the main medical theatres), were observed. This observation revealed improper technique being carried out by health professionals, which may have been directly correlated to patients’ pain and discomfort. Male patients undergoing flexible cystoscopies from October 2024 to January 2025 were asked to complete a questionnaire after their procedures. This questionnaire included the Numeric Pain Rating Scale (NPS) and the Visual Analog Scale (VAS). The following criteria had to be met for patients to be included in this study: the patients were male, aged 18 years or older, could comprehend the questionnaire fully, and had undergone a flexible cystoscopy on the same day. 

Questionnaires

The patients were asked to fill out a questionnaire with three questions during the study. First, the questionnaire asked whether the procedure was comfortable (to which they could answer yes or no). If they said that the procedure was not comfortable, the patients were asked to evaluate their level of discomfort using both the NPS and the VAS. Finally, the patients were asked how likely they would be to undergo the procedure again (Appendix A). A total of 80 patients fit the inclusion criteria and completed the questionnaire. Of these, 38 men were directly observed by healthcare professionals (including nurses and trainees) during urethral preparation prior to their flexible cystoscopy procedures. The remaining 42 men were directly observed by two consultants during urethral preparation prior to their flexible cystoscopy procedures. 

## Results

Using the VAS, 73.6% (28) of the 38 men (Group A) who were observed by healthcare professionals answered that they had experienced moderate pain or discomfort during their procedures, whereas 18.4% (seven) of these 38 men experienced mild pain during their flexible cystoscopy procedures (Table 1).

**Table 1:** Visual Analogue Scale comparing the results of the observed technique versus the standard technique

Visual Analogue Scale	Observed Technique (N=38)	Standard Technique (N=42)
	Pain or discomfort levels	Pain or discomfort levels
Mild	7 (18.4%)	31 (73.8%)
Moderate	28 (73.6%)	6 (14.3%)
Severe	3 (8%)	5 (11.9%)

In contrast, 14.3% (six) of the 42 men (Group B) who were observed by consultants answered that they had experienced moderate pain or discomfort during their procedures, whereas 73.8% (31) of these 42 men experienced only mild discomfort during the flexible cystoscopy once the improved technique was enforced (Table 1).

The number of men who experienced severe pain or discomfort was similar between the two groups: three for the observed technique and five for the standard technique (8% and 11.9%, respectively) (Table 1).

The results of the NPS (Table 2) coincided directly with those of the VAS and were therefore grouped together in the above table. On the NPS, scores of 1-3 were grouped with a ‘mild’ pain rating on the VAS, scores of 4-7 were grouped with a ‘moderate’ rating, and scores of 8-10 were grouped with a ‘severe’ rating (Table 1). These numbers are reflected directly in the VAS (Table 1).

**Table 2:** Numeric pain scale comparing the results of the observed technique versus the standard technique

Numeric Pain Scale	Observed Technique (N = 38)	Standard Technique (N=42)
	Pain or discomfort levels	Pain or discomfort levels
1 to 3	7 (18.4%)	31 (73.8%)
4 to 7	28 (73.6%)	6 (14.3%)
8 to 10	3 (8%)	5 (11.9%)

In the improved technique group, where Instillagel was properly applied, patients reported less pain and discomfort than in the observed technique group, where it was improperly applied. Once the proper technique of Instillagel application was made to the urethra prior to instrumentation with the flexible cystoscope, there was a shift in the pain and discomfort felt by the patients, and overall, satisfaction levels increased.

## Discussion

Outpatient cystoscopy is one of the most frequently performed procedures in urological practice. The relevant literature indicates that rigid cystoscopy, particularly in male patients, is associated with significant discomfort and often necessitates intravenous sedation [7]. The substantially uncomfortable and painful nature of this procedure has prompted numerous clinical trials aimed at enhancing patients’ tolerance of it [8,9]. In the mid-20th century, intraurethral instillation of anaesthetic gel was introduced for topical urethral anaesthesia. Subsequently, the use of amide-linked anaesthetics became a widely accepted approach to improve the tolerability of rigid cystoscopy, particularly in male patients [10].

In contrast, the use of flexible cystoscopy in male patients is associated with much less discomfort and better tolerability compared to rigid cystoscopy performed under local anaesthesia, as highlighted in the European Association of Urology (EAU) Guidelines. Thus, flexible cystoscopy has become the new gold standard cystoscopy procedure [11,12]. However, flexible cystoscopy also remains a generally uncomfortable procedure for patients to undergo [13,14].

Pre-procedural preparation for flexible cystoscopy closely parallels that of urethral catheterisation. Clinical guidelines recommend the routine use of lubricating gels in all patients undergoing such procedures to minimise the risk of urethral trauma and subsequent infection [15]. Gels like Instillagel containing a local anaesthetic (lidocaine hydrochloride, 5%) make the procedure less uncomfortable when applied correctly. To ensure the painless introduction of instruments, the entire urethra, including the external sphincter, must be coated with a film of lubricant and anaesthetised [16].

The use of topical local anaesthetic lubricating gel has been the mainstay and most widely accepted method for reducing discomfort and adverse symptoms associated with flexible cystoscopy [17]. The anaesthetic ingredient of Instillagel is lidocaine, which stabilises neuronal membranes and prevents the initiation and conduction of nerve impulses, thus affecting local anaesthetic action [16].

Of the 80 patients who participated in this audit, the most frequently reported outcome among those observed by a healthcare professional (Group A) was moderate to severe discomfort or pain during the insertion of the flexible cystoscopy (reported by 35 of 38 men). This was due to improper technique when placing the Instillagel into the urethra, rather than insufficient time allocated for the Instillagel to take effect.

According to the National Health Service (NHS) Greater Glasgow and Clyde (GGC) guidelines for placing Instillagel during Foley catheterisation for men (which is the same technique applied for placing a flexible cystoscope), Instillagel is slowly applied directly into the urethra and left for five minutes by holding the urethra closed between the thumb and the index (first) finger [6]. However, direct observation revealed two ways in which this guideline was not followed as stated and why attempting to follow it still would not be effective in reducing the pain and discomfort experienced by the men: (1) It was directly observed that the five-minute wait to allow the Instillagel to take effect was never achieved due to the sheer volume of patients requiring diagnostic or monitoring flexible cystoscopies. What was directly observed was that anywhere from 30 to 45 seconds was allowed for the Instillagel to take effect before the flexible cystoscope was inserted. (2) It was also directly observed that the Instillagel was not correctly applied. When placed by many of the healthcare professionals whom I directly observed, roughly 50% (~50%) of the Instillagel escaped the urethra (direct observation). When observing this phenomenon firsthand, it was clear why men were experiencing moderate to severe discomfort. 

The male urethra, on average, is 15-20 cm (6-9 in) [17]. However, if ~50% of the lubricant gel is escaping the urethra (due to poor technique, as directly observed), this implies that 7-10 cm (3-4.5 in) of urethra is not being adequately lubricated. Therefore, this phenomenon is likely to be the root cause of the significant pain and discomfort experienced by men during flexible cystoscopy (or, in many cases, urethral catheterisation). 

The improved technique employed involved pulling the penis a bit tauter while implementing the NHS GGC technique, which includes holding the urethra closed between the thumb and index finger. This allowed for the vast majority (roughly 90%, or 9-10 mL) of the Instillagel to span the length of the urethra. This technique was tested with the second group of 42 patients, and the results, as previously discussed, were expected.

One concern with holding the penis taut and expressing the Instillagel into the urethra is to ensure that it is not done too quickly. A few patients in Group B did mention a slight stinging sensation, which could be attributed to the rapid expansion of the urethra that occurs if the Instillagel is applied too quickly. However, overall, only low levels of discomfort were indicated by 73.8% (31 of 42) of the men in Group B. That said, practitioners should still be gentle and take their time when placing the Instillagel. 

One limiting factor to note was that this study included patients with a history of anxiety or hypersensitivity to pain. Those patients may be reflected in both Group A and Group B, as three and five patients, respectively, rated their pain and discomfort as severe.

## Conclusions

This QIP demonstrated that much of the pain and discomfort experienced by men undergoing flexible cystoscopy is largely attributed to inadequate urethral lubrication. The findings suggest that procedural discomfort is an unavoidable aspect of this operation that can be significantly influenced by preparation technique. Outcomes from Group B showed a clear and significant reduction in perceived pain and discomfort when enhanced lubrication methods were implemented. This highlights the direct impact that small, evidence-based adjustments in clinical practice can have on patient experience. The results reinforce the importance of prioritizing optimal urethral preparation prior to instrumentation. Overall, improved lubrication techniques represent a simple strategy to enhance patient comfort and procedural tolerance during flexible cystoscopy.

## Figures and Tables

**Figure 1:**
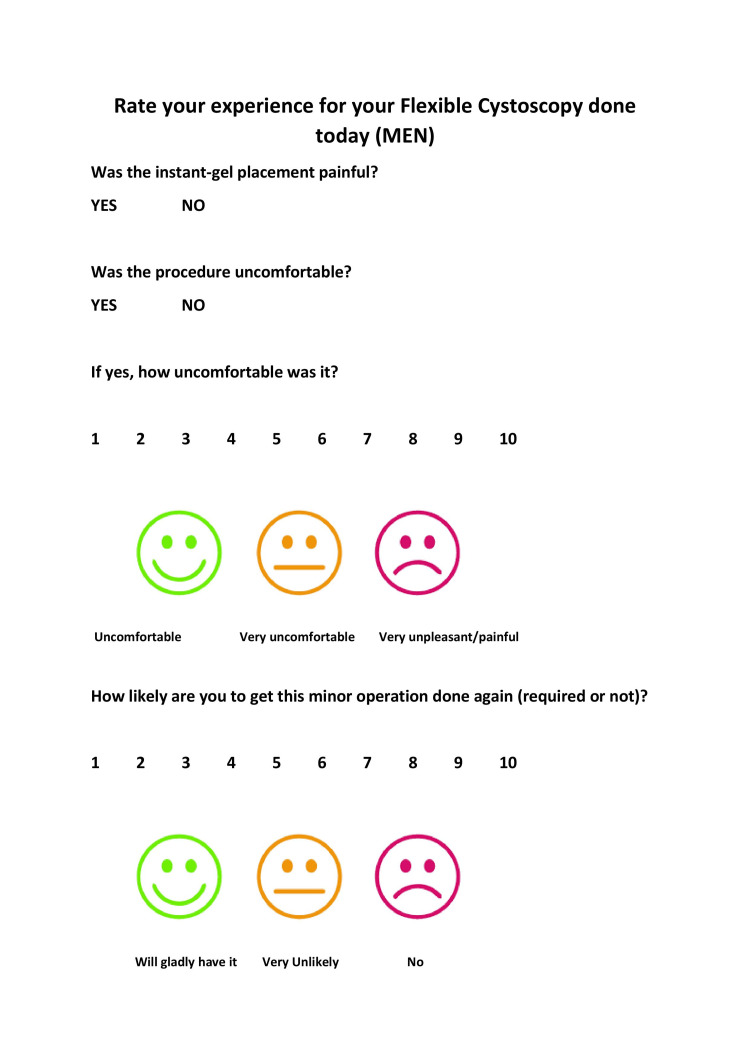
Survey for flexible cystoscopies in men

## Appendices

Appendix A 
